# Genome-Wide Identification and Characterization of Members of the *ACS* Gene Family in *Cucurbita maxima* and Their Transcriptional Responses to the Specific Treatments

**DOI:** 10.3390/ijms23158476

**Published:** 2022-07-30

**Authors:** Chaojie Wang, Wenling Li, Fangyuan Chen, Yaqian Cheng, Xin Huang, Bingxue Zou, Yunli Wang, Wenlong Xu, Shuping Qu

**Affiliations:** 1 Key Laboratory of Biology and Genetic Improvement of Horticultural Crops (Northeast Region), Ministry of Agriculture/Northeast Agricultural University, Harbin 150030, China; b19040104@neau.edu.cn (C.W.); s190401033@neau.edu.cn (W.L.); s200401046@neau.edu.cn (F.C.); s210402105@neau.edu.cn (Y.C.); s210402099@neau.edu.cn (X.H.); zoubingxue@kenfeng.com (B.Z.); wangyunli@neau.edu.cn (Y.W.); xwl@neau.edu.cn (W.X.); 2 College of Horticulture and Landscape Architecture, Northeast Agricultural University, Harbin 150030, China

**Keywords:** *ACS* gene family, *Cucurbita maxima*, sex determination, flower development

## Abstract

Ethylene biosynthesis and signal transduction play critical roles in plant sex differentiation. *ACS* (1-aminocyclopropane-1-carboxylic acid synthase) is a rate-limiting enzyme in ethylene biosynthesis. However, the understanding of the *ACS* gene family in *Cucurbita maxima* is limited. Here, we identified and characterized 13 *ACS* genes in the *C. maxima* genome. All *ACS* genes could be divided into three groups according to a conserved serine residue at the C-terminus. Thirteen *CmaACS* genes were found to be randomly distributed on 10 of the 20 chromosomes of *C. maxima*. The *ACS* gene exhibits different tissue-specific expression patterns in pumpkin, and four *ACS* genes (*CmaACS1*, *CmaACS4*, *CmaACS7*, and *CmaACS9*) were expressed specifically in both the female and male flowers of *C. maxima*. In addition, the expression levels of *CmaACS4* and *CmaACS7* were upregulated after ethephon and IAA treatments, which ultimately increased the number of female flowers, decreased the position of the first female flower and decreased the number of bisexual flowers per plant. These results provide relevant information for determining the function of the *ACS* genes in *C. maxima*, especially for regulating the function of ethylene in sex determination.

## 1. Introduction

The plant hormone ethylene is present in most plant tissues and plays important roles in the vegetative growth stage, reproductive growth stage, and mature stage of plants [1,2]. Ethylene is considered the main hormone affecting sex differentiation in Cucurbitaceae plants. This hormone determines the sex of each floral meristem by preventing the development of stamens and pistil primordia. It has been suggested that stamens or carpels may develop independently under different concentrations of ethylene due to different levels of sensitivity to the hormone [3]. Transformation of the meristem to a female flower requires high ethylene levels to prevent the development of the stamen primordium. However, low levels of ethylene lead to the transformation of the meristem into a male flower by preventing the development of carpel primordia, thus ensuring the normal development of flowers [4,5,6]. The ethylene-releasing agent ethephon has been widely used as a female flower induction reagent in melon [7], cucumber [8,9], and pumpkin [10]. At the same time, the use of ethylene-inhibiting treatments in melon [7], cucumber [11], and pumpkin was beneficial to the development of male flowers [7,11,12]. However, the role of ethylene in watermelon is opposite to that in other Cucurbitaceae species: ethephon treatment induces male flowers in watermelon [13,14].

In plants, ethylene is biosynthesized from methionine, which is converted to S-Adenosyl Methionine (ADOMet) under the action of ADOMet synthetase. ADOMet is converted to methylthioadenosine by ACS (EC4.4.1.14), and 1-aminocyclopropane-1-carboxylic acid (ACC) is the direct precursor of ethylene [15,16,17,18]. The conversion of SAM to ACC catalyzed by ACC synthase (ACS) is the rate-limiting step in the ethylene synthesis pathway, and ACS is the rate-limiting enzyme in this pathway [19,20,21,22]. ACS is encoded by polygene family members regulated by different plant development, environment, and hormone signals [23,24,25]. In addition, the nonfunctional ACS subtypes that interact with heterodimers between ACS proteins may regulate the enzyme activity of ACS through post-translational regulation [26]. Previous studies have shown that the orthologous genes *CmACS7*, *CsACS2*, *CitACS4,* and *CpACS27* control stamen stagnation and mutation in cucumber, melon, watermelon, and pumpkin, respectively, leading to transformation into andromonoecy from monoecy [27,28,29,30,31]. On the other hand, the loss of function of the orthologous genes *CsACS11* and *CmACS11* of cucumber and melon, respectively, completely block the path of female flower development, resulting in *androecy* [31,32]. Collectively, previous studies have shown that the ethylene synthase gene is directly related to sex determination in Cucurbitaceae.

Genome-wide analysis of the *ACS* gene family in most cucurbitaceous species has been reported. However, it has not been reported in *C. maxima* plants. There is still a lack of comprehensive understanding of *CmaACS* in pumpkin. Hence, a genome-wide comprehensive study was performed to identify putative pumpkin *ACS* family genes. Additionally, their phylogenetic relationships, chromosome distribution, gene structure, conserved motifs, homology, and cis-regulatory elements were characterized to obtain insights into *CmaACS* genes. Furthermore, the expression profiles in different tissues/organs and under the effects of numerous hormones have been extensively assessed. This study provides valuable information for further study of the cloning of key genes involved in ethylene synthesis and signal transduction pathways in pumpkin.

## 2. Results

### 2.1. Genome-Wide Identification of ACSs in Pumpkin

In this study, 13 *ACS* members were detected in the genome of pumpkin (Table 1). We named these *ACS* genes *CmaACS1–13* according to their gene IDs. Detailed information on these *ACS* genes, including their length, molecular weight (*M_w_*), isoelectric point (pI), and location, is shown in Table 1. The lengths of the amino acids of *CmaACS* proteins ranged from 442 aa to 549 aa, with the pIs varying from 5.61 to 8.96 and the *M*_w_s ranging from 49.81 kD to 60.27 kD. Furthermore, the predicted grand average of hydropathy (GRAVY) values of the *CmaACS* proteins ranged from −0.325 to −0.098, suggesting that they were hydrophilic. Moreover, subcellular location prediction revealed that five *CmaACS* proteins were positioned in the chloroplast, that four *CmaACS* proteins were positioned in the nucleus, and that four *CmaACS* proteins were positioned in the cytoplasm.

### 2.2. Multiple Sequence Alignment and Phylogenetic Analysis of CmaACS Gene Family

**Figure 1:**
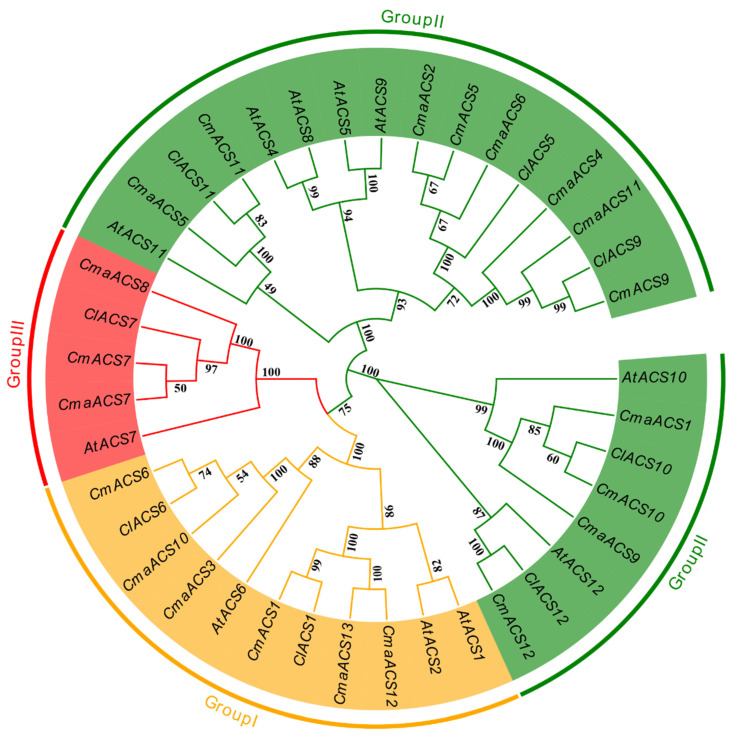
Phylogenetic relationship of 13 *ACSs* in *Cucurbita maxima* and other plants. All *ACSs* genes were divided into three groups based on the high bootstrap values and the phylogenetic tree’s topology.

Studies have shown that amino acid residues play a key role in enzyme activity [33]. The 13 *ACS* proteins identified were subjected to sequence alignment (Figure S1). All *ACS*s contained seven conserved regions found in the *ACS* of *Arabidopsis*, tomato, and other plant species. In *ACSs*, the amino acid residues in two regions (II and VII) are particularly conserved; therefore, changes in these amino acids may lead to different functions. To further evaluate the evolution of the *ACS* gene family, MEGA-X software was used to construct a phylogenetic tree of the full-length protein sequences of 41 *ACS* genes from three cucurbit species (melon, watermelon, and pumpkin) and *Arabidopsis* (Figure 1). The ACS proteins of *C. maxima* can be classified into three groups. Strikingly, the members of group II formed two different branches on the phylogenetic tree instead of clustering on the same branch, which may be due to their different motifs at the N-terminus [34] (Figure S1). *CmaACS1* and *CmaACS9* were closely matched to *AtACS10* and *AtACS12*, which are presumed to be amino acid transferases without ACS activity [25].

### 2.3. Gene Structure and Conserved Motif Composition of CmaACS Genes Family

**Figure 2:**
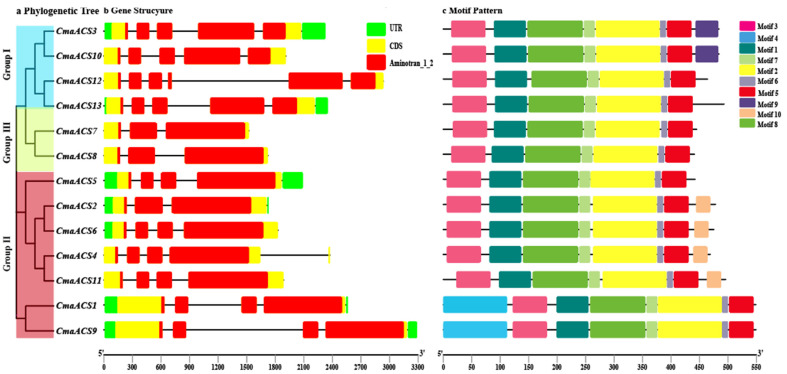
Phylogenetic relationships, gene structure, and architecture of conserved protein motifs in *CmaACSs*. (**a**) A phylogenetic tree based on the *CmaACSs* sequences. According to phylogenetic relationships, 13 *CmaACSs* were clustered into three groups (I-III) and represented with different colors. (**b**) The exon-intron structure of *CmaACSs*. Green boxes represent UTR regions, yellow boxes represent exons, black lines represent introns, and pink boxes represent Aminotran_1_2 domain. (**c**) The motif composition of *CmaACSs*. Different colored boxes display different motifs.

The exon-intron configurations of *CmaACS* genes were examined to acquire further insights into the probable structural evolution of the *CmaACS* family of genes. Our results show that the number of exons of the *CmaACS* genes ranged from three to five (Figure 2). This number is similar to that of the *ACS* family genes of *A. thaliana* [25], melon and watermelon [34]. For genes that are relatively closely evolutionarily related, the length and distribution of exons were similar (Figure 2b). We also investigated the full-length protein sequences of the 13 *CmaACSs* to identify their conserved motifs (Figure 2c) and identified ten motifs distributed among the *ACS* members, including six (motifs 1, 2, 3, 4, 5, and 6) aminotransferase domains (Table S3). Interestingly, *CmaACSs* in the same group often had similar motif compositions (Figure 2c); for example, motif 4 is specific to group III. The similar arrangement of motifs in the subgroups indicates that the protein structure is conserved within a particular subfamily. Overall, the results show that the members of a group have the same genetic structure, and their phylogenetic relationships remain unchanged. By studying the conserved motif constitution, gene structure, and phylogenetic relationship, we found that the stability of the group classification was robust, indicating that the *CmaACS* proteins have very conserved amino acid residues, that members of the same group may have similar functions, and that members of different groups have unique structures and motifs that may play different roles during plant growth and development.

### 2.4. Chromosomal Distribution and Synteny Analysis of CmaACS Genes

**Figure 3:**
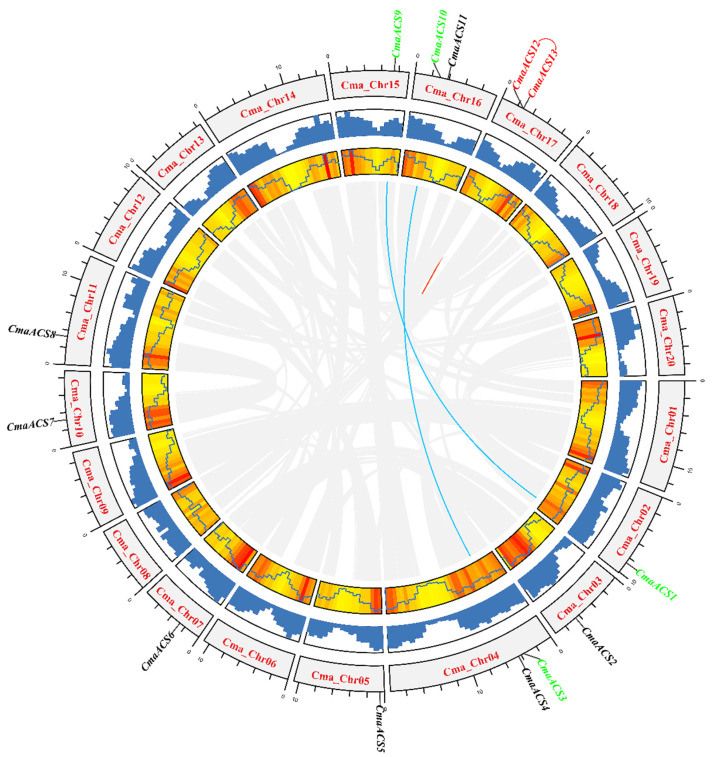
Chromosomal distribution of *CmaACS* genes. From the outside to the inside, the first circle represents chromosome coordinates; the second and third circles represent gene density distribution; blue or red lines connect gene pairs.

According to the chromosome annotation information of pumpkin, the identified pumpkin *ACS* gene was located on the chromosome (Figure 3). The results showed that there were two *ACSs* on chromosomes 4, 16, and 17 and one *ACS* each on chromosomes 2, 3, 5, 7, 10, 11, and 15, which were unevenly distributed. Gene duplication is ubiquitous and plays an important role in plant evolution. The built-in MCScanX software of TBtools (https://github.com/CJ-Chen/TBtools, accessed on 20 August 2021) was used to analyze the intraspecific collinearity of the *ACS* genes in pumpkin. Three pairs of collinear genes were detected, indicating that the six *CmaACS* genes were the result of duplication events (Figure 3). These results indicate that gene duplication promoted the increase in the number of *ACS* genes in the pumpkin genome to a large extent, and whole-genome duplication or fragment duplication may play a major role in driving this process.

**Figure 4:**
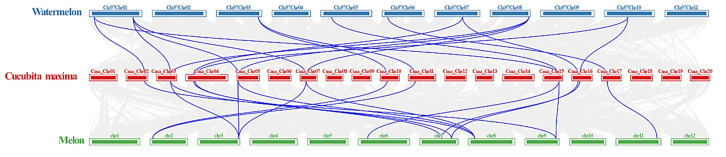
Synteny analysis of *ACSs* in watermelon, melon, and *C. maxima*. The blue lines represent the syntenic *ACS* pairs between the two genomes. The chromosome number is shown at the top of each chromosome.

To explore the evolution of the *ACS* gene family in Cucurbitaceae, we analyzed the homologous relationships between pumpkin, melon, and watermelon (Figure 4). Collinear analysis showed that there were a large number of linearly homologous *ACS* genes in pumpkin, melon, and watermelon. In addition, 16 pairs of genes in pumpkin and melon were collinear, 92.3% (12) of the pumpkin *ACS* genes had homologous genes in melon, 16 pairs of pumpkin and watermelon were collinear, and 92.3% (12) of the pumpkin *ACS* genes had homologous genes in watermelon. Taken together, these results showed that most of the *ACS* genes during the formation and evolution of pumpkin remained intact.

### 2.5. Cis-Elements in the Promoters of CmaACSs

**Figure 5:**
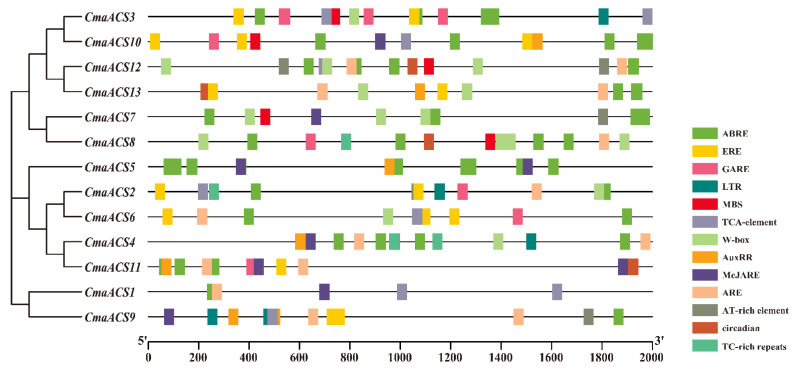
Cis-elements that are related to different stress and hormone responses in the putative promoters of *CmaACSs*. Cis-elements with similar functions are displayed in the same color. Different color boxes show different identified cis-elements.

Predicting the cis-elements in promoters that regulate gene expression is essential for improving the understanding of gene regulation [35]. We studied the 2000 bp sequence upstream of the initiation codon of each *CmaACS* gene, analyzed its cis-elements online via PlantCARE tools (http://bioinformatics.psb.ugent.be/webtools/plantcare/html/, accessed on 27 August 2021), and isolated the functional unknown elements and general transcriptional regulatory elements. As shown in Figure 5, various cis-elements related to stress and plant hormone responses were detected in these *ACS* genes, such as abscisic acid-responsive elements (ABREs), gibberellin-responsive elements (GARE motifs), salicylic acid-responsive elements (TCA elements), MeJARE-responsive elements (MeJAREs), auxin-responsive elements (AuxRRs), and ethylene-responsive elements (EREs and W-boxes). Stress-related elements, including AREs, LTRs, MBSs, AT-rich-elements, circadian-related elements, and TC-rich repeats, were present in a small number of *ACS* genes. Most hormone- and stress-responsive elements were present upstream of a specific small number of *CmaACS* genes, suggesting that these elements play key roles in the response to hormones and in stress response mechanisms.

### 2.6. Expression Analysis of CmaACSs in Pumpkin Tissue

**Figure 6:**
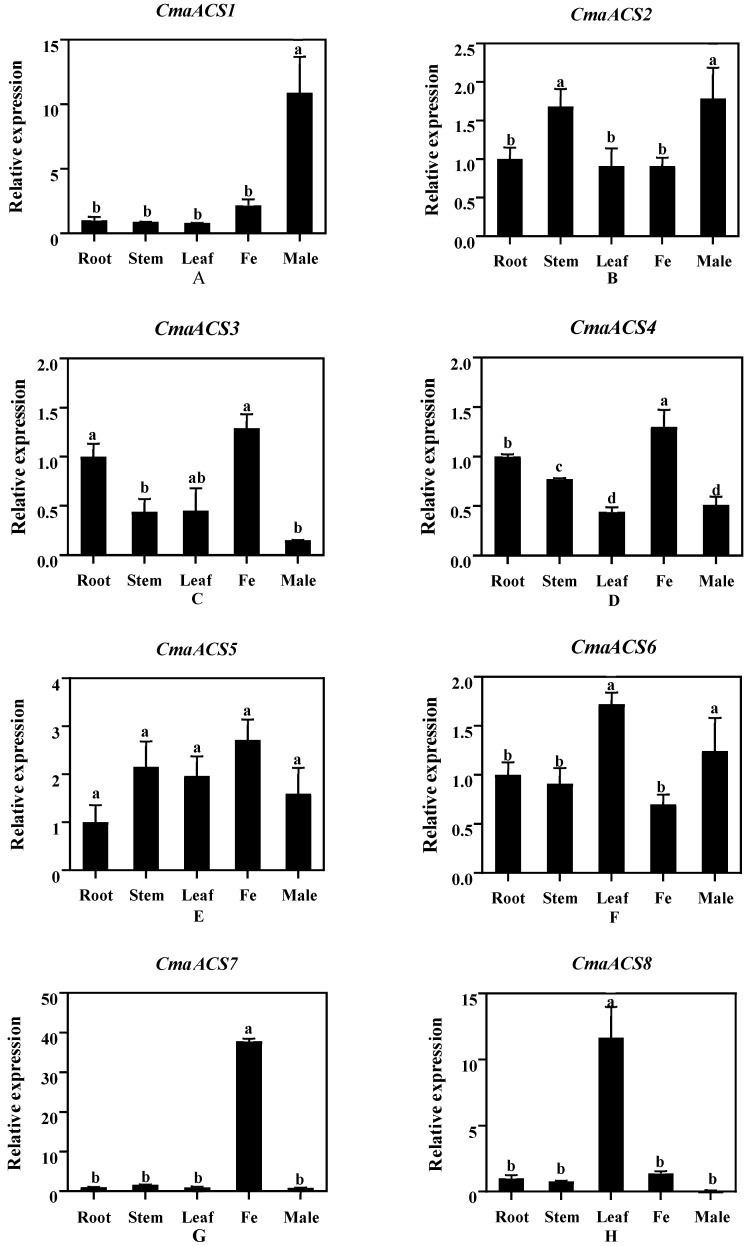

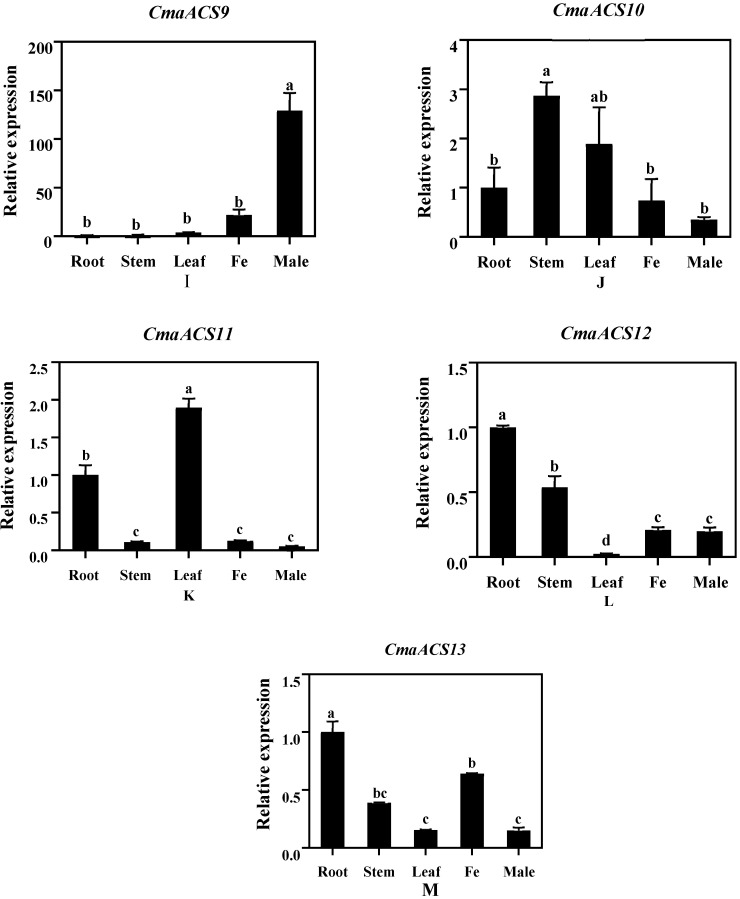
Expression patterns of *CmaACS* genes in different tissues. (**A**–**M**) Relative expression levels of *ACS* gene family members in different tissues. Means followed by a different letter in each column are statistically different by SSR’s test at *p* > 0.05.

To understand the potential function of specific *ACS* isozymes in pumpkin, we analyzed the tissue-specific expression patterns of the *CmaACS* genes in different tissues of pumpkin. The results showed that the 13 *CmaACS* genes were differentially expressed in the roots, stems, leaves, female flowers, and male flowers. *CmaACS1*, *CmaACS4*, *CmaACS7,* and *CmaACS9* were specifically and highly expressed in the male and female flowers. Although *CmaACS3* and *CmaACS5* were highly expressed in the female flowers, the expression was not significantly different from that in the other organs. *CmaACS8* and *CmaACS11* were highly expressed in the leaves, *CmaACS12* and *CmaACS13* were specifically expressed in the roots, and the expression of the other genes was not significantly different across the various organs (Figure 6). Interestingly, a relatively strong expression of four *CmaACSs* (*CmaACS1*, *CmaACS4*, *CmaACS7*, and *CmaACS9*) was observed in the female and male flowers of *subgynoecious* pumpkin, suggesting that the different expression patterns were associated with flower development.

### 2.7. Expression Profiles of the CmaACSs of C. maxima at Different Flower Developmental Stages

**Figure 7:**
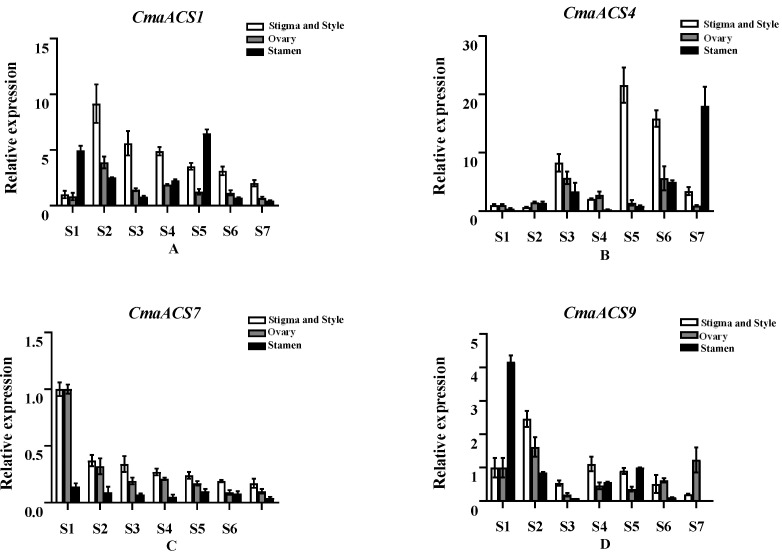
Comparison of expression patterns of *CmaACS1* (**A**), *CmaACS4* (**B**), *CmaACS7* (**C**)*,* and *CmaACS9* (**D**) at different flower development stages.

To investigate whether *CmaACS1*, *CmaACS4*, *CmaACS7,* and *CmaACS9* were related to pumpkin sex determination, qRT-PCR was used to measure their expression at different stages (S1–S7) in the stamens, ovaries, stigmas, and styles. As shown in Figure 7A, the expression in the ovaries, stigmas, and styles of *CmaACS1* tended to increase first but then decrease during flower development; the expression level at S2 was the highest. The *CmaACS1* expression in the stamens showed a bimodal trend, peaking at S1 and S5, and the highest expression level among the stamens was recorded at S5. These *CmaACS1* expression trends indicate that *CmaACS1* may play an important role in the early development of stamens, stigmas, and styles. The expression of *CmaACS4* in the stigmas, styles, ovaries, and stamens showed a bimodal trend during flower development. The expression of *CmaACS4* was the highest at S5 in the stigmas and styles and was the highest at S3 in the ovaries. The expression level at S7 was the highest in stamens (Figure 7B); it is speculated that *CmaACS4* is related to pistil and stamen maturation. The expression of *CmaACS7* in the ovaries, stigmas, and styles was significantly reduced during flower development, and the expression level was highest at S1. The expression of *CmaACS7* in the stamens was very low at different stages of pumpkin stamens. Across S1–S7, the expression of *CmaACS7* in the ovaries, stigmas, and styles was significantly higher than that in the stamens, and the expression in S1 was significantly higher than that in the stamens. The trend of *CmaACS7* expression shown in Figure 7C indicates the important role of *CmaACS7* in early sex differentiation. The expression of *CmaACS9* in the stigmas, styles, ovaries, and stamens showed a bimodal trend. Moreover, *CmaACS9* was highly expressed in the S2 phase of the ovaries, stigmas, and styles, and *CmaACS9* was specifically and highly expressed in the S1 phase of the stamens (Figure 7D), which suggests that *CmaACS9* may be involved in the early development of stamens.

### 2.8. Expression Profiles of CmaACSs in Pumpkin under Phytohormones and Ethylene Inhibitors Treatment

**Figure 8:**
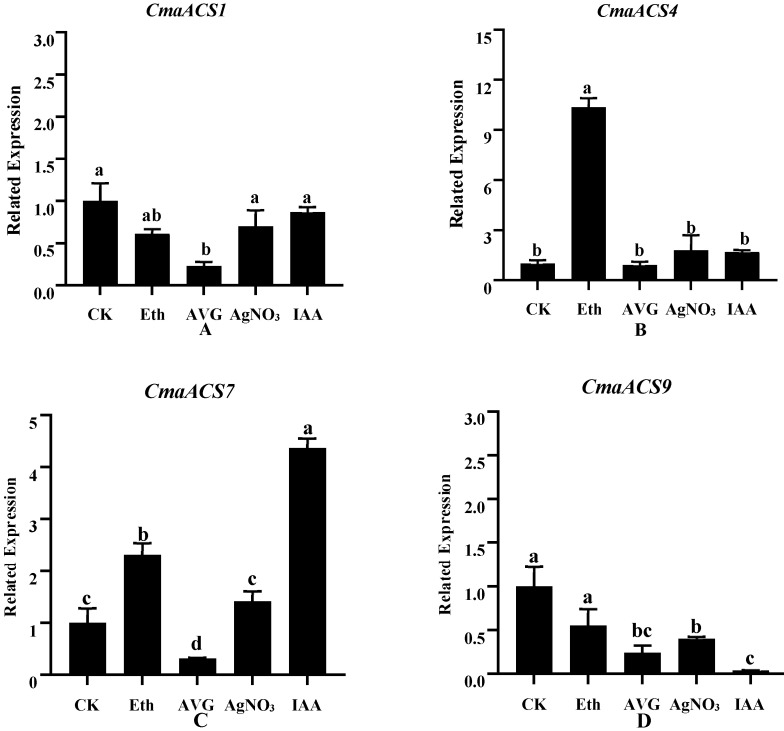
Comparison of expression patterns of *CmaACS1* (**A**), *CmaACS4* (**B**), *CmaACS7* (**C**)*,* and *CmaACS9* (**D**) Under Different Hormone Treatments. Means followed by a different letter in each column are statistically different by SSR’s test at *p* > 0.05.

To reveal the possible role of *CmaACSs* after Ethephon (an ethylene-releasing agent), AVG (an inhibitor of ethylene biosynthesis), AgNO_3_ (an inhibitor of ethylene biological response), and IAA treatment, gene expression analysis of four selected *ACSs* were performed using quantitative RT-PCR. In the treatment with exogenous hormones, ethephon and IAA increased the number of female flowers per plant in ‘2013-12’, decreased the position of the first female flower, and reduced the number of bisexual flowers per plant (Table 2). Compared with that of the ‘2013-12’ control, the expression level of *CmaACS1* after AVG treatment was significantly reduced, while ethephon, IAA and AgNO_3_ had no significant effect on the expression of *CmaACS1* (Figure 8A). As shown in Figure 8B, compared to the ‘2013–12’ control, the expression level of *CmaACS4* significantly increased after ethephon treatment. Compared with that of the ‘2013-12′ control, the expression level of *CmaACS7* after ethephon and IAA treatment significantly increased, while the mRNA expression level of *CmaACS7* significantly decreased after AVG treatment. AgNO_3_, an ethylene biosynthesis inhibitor, had no significant effect on the expression of *CmaACS7* (Figure 8C). As shown in Figure 8D, compared with that of the ‘2013-12′ control, the expression level of *CmaACS9* after treatment with ethephon, AVG, AgNO_3_ and IAA was significantly reduced. Taken together, these results indicate that *CmaACSs* may play crucial roles in ethylene synthesis and signal transduction pathways as well as in the sex determination of pumpkin.

## 3. Discussion

*ACS* is one of the rate-limiting enzymes in the process of endogenous ethylene biosynthesis and is very important for the regulation of endogenous ethylene biosynthesis. Members of the *ACS* polygene family have been cloned and identified from various plant species. The results of genome screening showed that there are 12 *ACS* genes in *A. thaliana* [25], 14 *ACS* genes in bananas [36], 9 *ACS* genes in tomatoes [21], and 10 *ACS* genes in grapes [37] suggesting that gene expansion, diversification, and functional redundancy contributed to the evolution of the gene family [33,38]. The number of *ACS* genes is similar among Cucurbitaceae species. The number of ACS genes were increased in pumpkin compared to cucumber [33], melon, and watermelon [34]. The intraspecific collinearity of the *ACS* genes in a pumpkin was analyzed by TBtools built-in MCScanX software (https://github.com/CJ-Chen/TBtools, accessed on 20 August 2021), and three pairs of collinear genes were detected (Figure 3). Among them, the amino acid sequence identity of *CmaACS2* and *CmaACS6* reached 90.79%, the amino acid sequence identity of *CmaACS10* and *CmaACS3* reached 89.69%, and the amino acid sequence identity of *CmaACS13* and *CmaACS12* reached 85.94% (Table S2). This shows that certain isozymes encoded by the *ACS* genes in pumpkin are the results of gene duplication or diversification. Previous studies have shown that the chromosome distribution of the *ACS* gene is uneven. In this study, we identified and characterized 13 *ACS* genes in the *C. maxima* genome; these genes were distributed on 10 chromosomes, and there were two *ACS* genes on chromosomes 4, 16 and 17. Consistent with the results of previous studies, we found that the *ACS* gene is unevenly distributed on multiple chromosomes. The *ACS* gene structure analysis, nucleotide sequence and motif arrangement of pumpkin are consistent with those in previous studies on *AtACS* and *CsACS* genes in *Arabidopsis*, melon, and watermelon [25,34]. *ACS* proteins can be divided into three categories based on the sequence characteristics of the C-terminal [25,39]. According to this principle, all the identified *CmaACS* proteins were clustered into three categories, showing high similarity with the proteins in *A. thaliana*, melon, and watermelon [25,34]. In addition, the phylogenetic results are also supported by the gene structure and motif distribution. *ACS* genes clustered into distinct clades are associated with distinct motifs that are regulatory sites for post-transcriptional regulatory mechanisms [40]. *CmaACS1* and *CmaACS9* clustered together with *AtACS10* and *AtACS12. AtACS10,* and *AtACS12* are ACS-like, which are considered to be amino acid transferases without ACS activity [27]. *CsACS10* and *CsACS12* clustered with *AtACS10* and *AtACS12* in cucumber and were abundantly expressed in cucumber leaves, cotyledons, and tendrils. Enzyme activity assays showed that *CsACS1-2* and *CsACS6* were active, *CsACS9*, *CsACS10,* and *CsACS12* were inactive, and denatured *CsACS1-2*, *CsACS2,* and *CsACS6* were inactive. Combined with a comparison of amino acid sequences, their studies suggest that amino acid conservation in invariant residues and conserved motifs of CsACS proteins is important for their in vitro ACS activity [33]. In the present study, we found that *CmaACS1* and *CmaACS9* were highly expressed in male flowers, and there were significant changes in the conserved residues and domain amino acids of ACS proteins and transaminases (Figure S1). Therefore, further experiments are needed to determine whether *CmaACS1* and *CmaACS9* have ACC-producing activity.

Members of the *ACS* gene family exhibit different tissue expression patterns in different plant species, suggesting that these genes have potentially different functions in plant development. In this study, 13 *CmaACS* genes were differentially expressed in the roots, stems, leaves, female flowers, and male flowers. *CmaACS7* was highly expressed specifically in the female flowers (Figure 6); *CmaACS7* was expressed specifically in S1 female flowers; and its expression in the ovaries, stigmas, and styles was higher than that in the stamens (Figure 7C). The homologous genes *CmACS7* and *ClACS7* in the third group are directly associated with andromonoecy [31,32,41,42], which suggests that *CmaACS7* is also involved in female development. *CmaACS4* is highly expressed in females. *CmaACS4* is homologous to *CmACS11* and *ClACS11*, and both *CmACS11* and *ClACS11* are involved in female flower development [31,32,34]. The expression of *CmaACS4* was the highest at S5 in the stigmas and styles, the highest at S3 in the ovaries, and the highest at S7 in the stamens (Figure 7B). It is thus speculated that *CmaACS4* is also involved in female flower development. *CmaACS1* and *CmaACS9* are homologous to *CmACS11* and *ClACS11*, but *CmaACS1* and *CmaACS9* are highly expressed in male flowers (Figure 6). It was found that the double peaks of *CmaACS1* expression in the stamens appeared at S1 and S5, and the expression at S5 was higher than that at S1. The expression level of *CmaACS9* in the stamens at S1 was significantly higher than that in other tissues (Figure 7A,D), indicating that these genes are involved in male flower development. It is speculated that homologous genes may play different functions in different plant species.

The use of ethylene and ethylene inhibitors may directly affect the expression of sex-determining genes [43,44]. The expression of *CitACS1* and *CitACS4* was upregulated after watermelon was treated with ethephon, while the expression of *CitACS2* and *CitACS3* was downregulated; in *A. thaliana*, the expression of *AtACS2~7* and *AtACS9~12* was upregulated in response to IAA, and that of *AtACS4*, *AtACS6,* and *AtACS7* was upregulated in response to ethephon [25,45,46]. After applying AgNO_3_, we found that the expression of *CsACS2* in *gynoecious* tissues was higher than that in *subgynoecious* tissues. After AgNO_3_ treatment, the expression level of *gynoecious* and *subgynoecious* proteins increased [47]. In our study, both ethylene and IAA treatments could increase the number of female flowers and reduce the node position of female flowers. After the application of ethylene, the expression of *CmaACS4* and *CmaACS7* in pumpkin increased. Analysis of cis-elements showed that all *CmaACS* genes except *CmaACS1*, *CmaACS5,* and *CmaACS8* contained a large number of EREs (Figure 5). This difference in sequence structure suggests that the inducible expression of *CmaACSs* by ethylene is associated with the occurrence of ethylene-inducible regulatory elements in their promoter sequences (Figure 5). After the application of IAA, the expression of *CmaACS7* increased, the number of female flowers increased, the number of bisexual flowers decreased, and the first female flower appeared earlier. IAA treatment can promote ethylene production and ethylene-related gene expression [10,48,49,50]. Therefore, we speculated that IAA may affect the sex determination of pumpkin by inducing the expression of *CmaACS7* and indirectly affecting the production of ethylene. Moreover, it was observed that the expression of *CmaACS9* was downregulated after ethylene treatment, and the increase in exogenous ethylene content inhibited the production of male flowers. It is thus presumed that *CmaACS9* is closely related to the production of male flowers. The application of the ethylene biosynthesis inhibitor AVG and the ethylene sensing inhibitor silver nitrate has been shown to delay the transition to female flowers and reduce the number of female flowers per plant [7,51]. Our study also confirmed this result. AVG is a non-specific competitive inhibitor of *ACS* and has been shown to block ethylene biosynthesis [52,53,54]. *ACS* genes are regulated by ethylene through negative feedback or feedforward mechanisms. In this study, the expression levels of *CmaACS1*, *CmaACS4*, *CmaACS7,* and *CmaACS9* were decreased after AVG treatment (Figure 8), so it is speculated that the *ACS* genes may be regulated by feedback of the ethylene mechanism. Silver ions were found to block ethylene sensing, and their effects were associated with inhibition of ethylene metabolism [55]. Furthermore, silver nitrate has nonspecific and undesirable off-target effects [54]. We verified the expression changes of *ACS* genes after silver nitrate treatment. The expressions of *CmaACS1*, *CmaACS4,* and *CmaACS7* were not significantly different compared with the control group, and only the expression of *CmaACS9* was decreased (Figure 8). The results showed that silver nitrate treatment only affected the expression of some *ACS genes*. It was speculated that the ethylene sensor may play a more important role in the sex determination of pumpkins. The direct relationship between the expression of *CmaACSs* and sex determination is currently unclear. Therefore, in situ hybridization should be used to further study the expression kinetics of *CmaACSs* at specific developmental stages.

## 4. Materials and Methods

### 4.1. Prediction and Identification of CmaACSs in C. maxima

To identify potential *CmaACSs* in pumpkin, a hidden Markov model (HMM) of the transaminase I and II domain (PF00155) was obtained from the Pfam protein family database and used to identify the putative *ACS* sequence with default parameters by the BlastP method. Searches for *ACSs* were performed using HMMER 3.0 (Howard Hughes Medical Institute: Cambridge, MA) (E-value ≤ 10^−5^). According to a recently published study, the amino acid sequence of the protein encoded by the *AtACS*, *CmACS,* and *ClACS* genes was retrieved from the TAIR (http://www.arabidopsis.org/ accessed on 5 June 2020), melon and watermelon (http://cucurbitgenomics.org/ accessed on 7 June 2020) genome database [25,56,57]. Afterwards, all the putative ACS amino acid sequences of pumpkin were obtained from the cucurbit genomic database (http://cucurbitgenomics.org/ accessed on 24 May 2020). Furthermore, candidate proteins were confirmed with SMART (http://smart.embl.de/ accessed on 10 June 2020) [58] and Pfam databases (http://pfam.xfam.org/ accessed on 15 June 2020) [59]. The information on molecular weights, isoelectric points, and predicted subcellular location was obtained from the ExPasy website (http://web.e-xpasy.org/protparam/ accessed on 15 August 2020) [60]. The WoLF PSORT II (https://www.genscript.c-om/wolf-psort.html accessed on 15 August 2020) online server was used to predict subcellular locations [61].

### 4.2. Sequence Analysis and Structural Characterization

Multiple Expectation Maximization for Motif Elicitation (http://meme-suite.org accessed on 17 August 2020) was used to analyze the conserved *C. maxima* protein sequences. For conserved motifs discovery, the maximum number of motifs was set to 10. The classic mode and motif width of 6–200 amino acids were selected. TBtools (V 1.098) software (https://github.com/CJ-Chen/TBtools, accessed on 20 August 2021) was used to display the *CmaACSs* gene structures [62]. The amino acid sequence encoded by the *ACS* gene of pumpkin in the database was analyzed by ClustalW with default parameters [63].

### 4.3. Phylogenetic Analysis

To investigate the phylogenetic relationships of *CmaACS* genes, 13 *CmaACSs* and *ACS* sequences of other species were used for phylogenetic analysis. The phylogenetic tree was constructed through the Neighbor–Joining (NJ) method under the parameters of the Poisson model, complete deletion, and 1000 bootstrap replicates using MEGAX software v.10.1.8 (Mega Limited, Auckland, New Zealand) (https://www.megasoftwar-e.net/ accessed on 2 April 2021). The results were formatted for display using the Evolview V3 (https://www.evolgenius.info/evolview/ accessed on 2 April 2021) [64].

### 4.4. Promoter Sequence Analysis

The sequences of 2 kb upstream from the start codon were downloaded from Genoscope and defined as the promoter of each *CmaACSs*. Then, PlantCARE [65] was used to predict cis-elements and display them using Tbtools [62].

### 4.5. Analysis of the Collinearity and Selection Pressure of the ACS Gene Family

One Step MCScanX of TBtools was used to detect gene duplication events. The gene density of the genomes, the position of the *CmaACSs* on the chromosome, and the gene replication relationship are displayed by the Advanced Circos of Tbtools. TBtools built-in McScanX software were used to analyze the *ACS* genes of *A. thaliana*, watermelon, melon, and pumpkin with collinearity, and use Circos (http://circos.ca/software/download/cir-cos/ accessed on 2 June 2021) to draw the relationship diagram. Ka/Ks_Calculator of TBtools was used to calculate non-synonymous (ka) and synonymous (ks) substitution [62].

### 4.6. Expression Pattern of CmaACSs in C. maxima

The experiment was carried out with the *subgynoecious* inbred line ‘2013-12’. The seeds of ‘2013-12’ were germinated at 28 °C in the dark for 36 h after being treated with 55 °C water for 24 h and then transplanted to greenhouse breeding plots at Northeast Agricultural University. Three-leaf stage seedlings were transferred to a greenhouse, and the required irrigation and fertilizer were applied under natural photoperiodic conditions in the spring of 2017. Three biological replicates of plant tissues, including roots, stems, leaves, female flowers, male flowers, and flowers of different developmental stages, were sampled and frozen in liquid nitrogen to extract RNA.

In order to study the effects of phytohormones and ethylene inhibitor treatments on *CmaACS* gene expression, we selected *CmaACS* genes specifically expressed in flowers for further qRT-PCR analysis. The shoot apices of ‘2013-12’ at the four-true leaf stage were sprayed continuously with 100 mg/L 2-chloroethylphosphonic acid (Ethephon) (an ethylene-releasing agent), 100 mg/L Aminoethoxyvinyl glycine (AVG) (an inhibitor of ethylene biosynthesis), 200 mg/L AgNO3 (an inhibitor of ethylene biological response), and 200 mg/L indoleacetic acid (IAA) for 4 times (continuously) once per day, and each treatment was composed of 15 plants and three biological replicates. After treatment for one day, the shoot apices of ‘2013–12’ that had been subjected to the different chemical solutions were selected. All treated tissue samples were immediately frozen in liquid nitrogen and stored at −80 °C for subsequent analysis.

### 4.7. RNA Extraction and Gene Expression Analysis

Total RNA was extracted using the Trizol method as described by Ma et al. [66]. All RNA was analyzed by agrose gel electrophoresis and then quantified with a Nanodrop ND-1000 spectrophotometer. First-strand cDNA was synthesized from the total RNA by using a PrimeScript RT reagent kit (Takara Biomedical Technology, Dalian, China) according to the manufacturer’s instructions. The quantitative RT-PCR was carried out with the Roche Lightcyler^®^ 480 instrument (Roche Diagnostics Nederland BV, Almere, Holland) using SYBR Green chemistry. The β-actin gene was used as an internal control. The reaction was carried out as follows: 95 °C for 30 s, followed by 40 cycles of 95 °C/10 s, 60 °C/30 s. Each reaction was performed in biological triplicates and the data from real-time PCR amplification was analyzed using 2^−^^△△^CT method [67]. The sequences of the primers used in this study are shown in detail in Table S1.

## 5. Conclusions

In this study, 13 *CmaACS* genes were characterized, their expression profiles were investigated, and their possible functions were discussed. The results showed that *CmaACS1*, *CmaACS4*, *CmaACS7,* and *CmaACS9* were highly expressed in both female and male flowers of pumpkin, and their expression patterns were analyzed at different flower development stages and in response to different treatments. It is speculated that *CmaACS1* has little effect on sex differentiation but that *CmaACS4*, *CmaACS7,* and *CmaACS9* may be involved in sex differentiation. Our study provides comprehensive information on *CmaACS* genes and provides relevant information for understanding the function of ethylene in sex differentiation.

## Figures and Tables

**Table 1:** The characteristics of 13 *ACSs* in *Cucurbita maxima*.

Gene	Gene ID	Chromosomal Localization	CDS Length (bp)	Protein Length (aa)	*M_w_* (kDa)	pI	Subcellular Localization
*CmaACS1*	*CmaCh02G015170.1*	Chr02: 8650601~8653154	1650	549	60.14	5.65	Chloroplast
*CmaACS2*	*CmaCh03G008840.1*	Chr03: 6510606~6512326	1437	478	53.80	7.98	Nucleus
*CmaACS3*	*CmaCh04G007260.1*	Chr04: 3694549~3696868	1455	484	54.31	6.61	Cytoplasm
*CmaACS4*	*CmaCh04G008430.1*	Chr04: 4336601~4338975	1410	469	53.05	8.96	Nucleus
*CmaACS5*	*CmaCh05G001180.1*	Chr05: 508990~511072	1329	442	49.88	9.42	Cytoplasm
*CmaACS6*	*CmaCh07G006250.1*	Chr07: 2713056~2714884	1428	475	53.48	8.43	Nucleus
*CmaACS7*	*CmaCh10G007020.1*	Chr10: 3155561~3157085	1338	445	49.92	5.86	Cytoplasm
*CmaACS8*	*CmaCh11G006760.1*	Chr11: 3270641~3272363	1326	441	49.81	5.61	Chloroplast
*CmaACS9*	*CmaCh15G011790.1*	Chr15: 7462460~7465739	1650	549	60.27	5.76	Chloroplast
*CmaACS10*	*CmaCh16G005820.1*	Chr16: 3012078~3013989	1455	484	54.22	7.60	Cytoplasm
*CmaACS11*	*CmaCh16G007300.1*	Chr16: 3845420~3847307	1491	496	56.18	8.57	Nucleus
*CmaACS12*	*CmaCh17G004550.1*	Chr17: 2713968~2716904	1395	464	52.53	6.90	Chloroplast
*CmaACS13*	*CmaCh17G004560.1*	Chr17: 2729331~2731675	1482	493	55.90	6.77	Chloroplast

CDS—coding DNA sequences, bp—base pair, *M_w_—*molecular weight, pI—isoelectric points.

**Table 2:** Effects of phytohormones and ethylene inhibitors treatments on sex differentiation of ‘2013-12′.

Treatments	Number of Female Flowers Per Plant	First Female Flower Position	Number of Bisexual Flowers Per Plant
Ethephon	14.8 ± 0.6a	5.7 ± 0.5c	0.5 ± 0.2b
IAA	14.3 ± 1.2a	6.8 ± 1.4c	1.3 ± 0.4b
AVG	9.8 ± 0.8b	11.3 ± 0.9b	5.4 ± 0.9a
AgNO_3_	6.5 ± 0.2c	12.9 ± 1.0a	6.4 ± 0.9a
CK	10.2 ± 0.8b	10.1 ± 1.1b	5.2 ± 1.1a

Note: Values are the mean (± standard error) of 45 plants. Means followed by a different letter in each column are statistically different by SSR’s test at *p* > 0.05.

## Data Availability

Not applicable.

## Supplementary Materials

The following supporting information can be downloaded at: https://www.mdpi.com/article/10.3390/ijms23158476/s1.
