# Supplementary material for: Genome-Wide Identification and Characterization of Members of the ACS Gene Family in Cucurbita maxima and Their Transcriptional Responses to the Specific Treatments
Source: Int J Mol Sci. 2022 Jul 30;23(15):8476. doi: 10.3390/ijms23158476 (PMC9369044; doi:10.3390/ijms23158476)
Supplement: Supplementary file 1 [file ijms-23-08476-s001.zip › Supplementary figures.pdf]

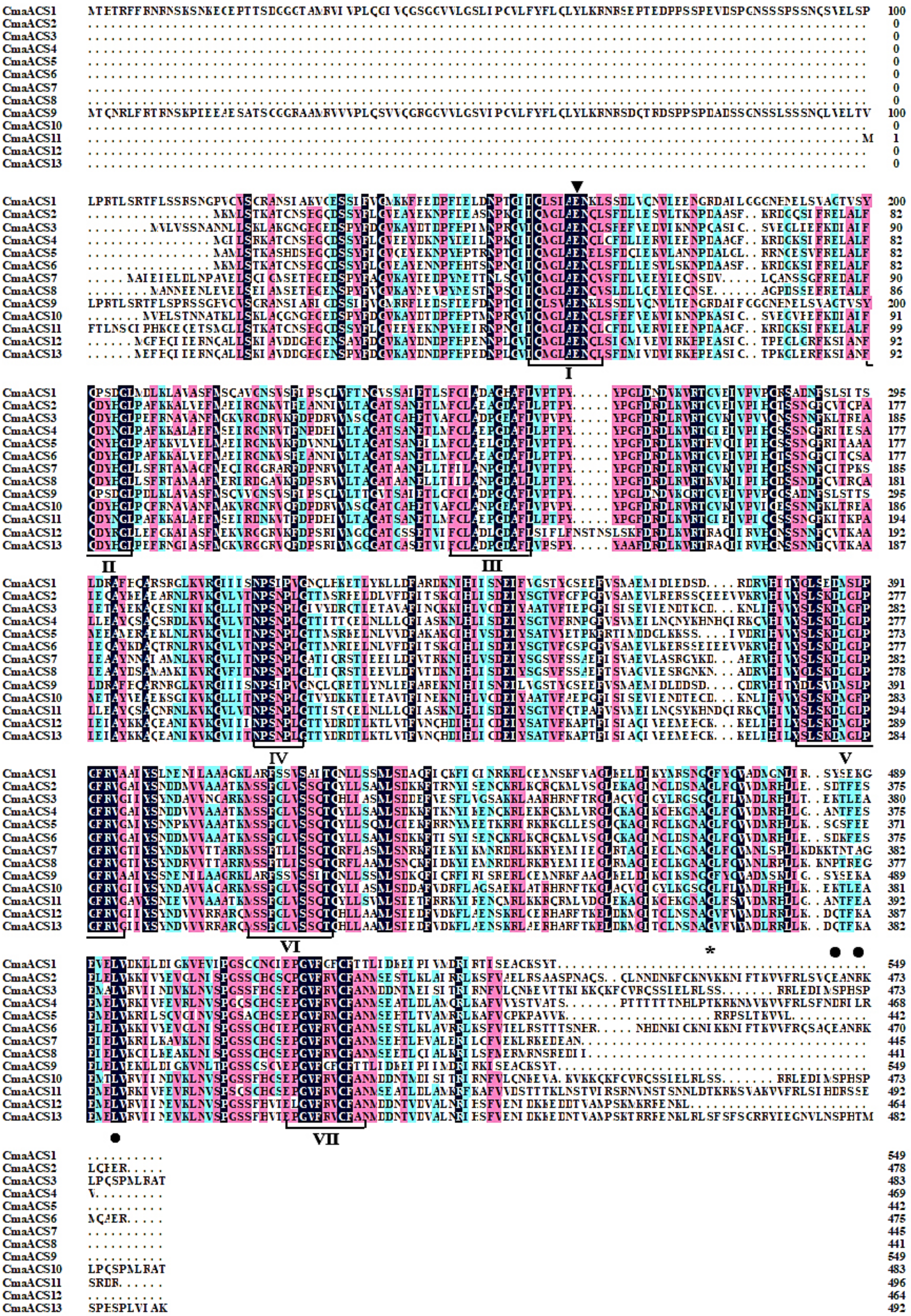

Figure S1. Amino acid sequence alignment of *CmaACS* genes. The conserved glutamate residue (E) marked with an arrowhead is involved in substrate specificity. Ser residues implicated in calcium-dependent protein kinase (CDPK) and mitogen-activated protein kinase (MPK6) phosphorylation are marked by asterisks and a black dot, respectively. The seven highly conserved regions (I–VII) among all ACC synthases are underlined.
